# Telehealth research

**Published:** 2012-06-15

**Authors:** Ombarish Banerjee, Maria Tanner

**Affiliations:** Oxleas NHS Foundation Trust, England; Oxleas NHS Foundation Trust, England

**Keywords:** telehealth, COPD

## Abstract

**Aims:**

Oxleas Telehealth is piloting the effectiveness of remotely monitoring Tier 3 COPD^1^ patients to reduce the hospital admissions and enable community nurses to reach more patients. Setting alert thresholds for clinical readings, which indicate an ill patient if exceeded, is crucial to remote monitoring. Hitherto, thresholds have been set from national guidelines. Patient specific adjustments are then made upon periodic qualitative assessments of progress. This causes the following problems:

- Excessive red alerts taking long time to clinically triage
- No standardised method for adjusting thresholds
- Anticipated operational issues when upscaling

Oxleas is researching a quantitative method for setting thresholds by statistically analysing daily variation in patients’ clinical measurements, to differentiate between common place variation in physiology, and special case variation due to ill-health.

**Potential benefits:**

- Reduced nurse time spent triaging data and contacting patients with red alerts but well
- Freed up nursing time to deliver more community care to patients
- Efficient method for threshold setting when upscaling

**Cycle 1 method:**

For each patient, pulse, oxygen and blood pressure readings were collated for the two preceding months. Arithmetic means were calculated for each. Spread analysis confirmed a bell shaped distribution. Alert thresholds were set three standard deviations from mean for each parameter. Exceptions were made for variation indicating good health (e.g. high oxygen levels). Episodes of abnormal readings were compared to clinical notes to find correlation with episodes of illness.

**Cycle 1 result:**

Correlation revealed 100% sensitivity, where all episodes of illness produced measurements exceeding thresholds. Specificity was poor (85% for oxygen, 60% for pulse and 52% for blood pressure) with numerous false positive readings. Of false positives, 61% occurred due to technical error (mostly faulty batteries or poor user technique). False positives for non-technical reasons were 78% for blood pressure, 57% for pulse and 20% for oxygen.

**Cycle 2 method:**

This was performed to audit technical error. From cycle 1 results, eliminating technical error would improve specificity to 86% for oxygen, 73% for pulse and 63% for blood pressure. Drop down menus were installed on the triage software to code for:

1. Number of abnormal measurements daily
2. Number of technical errors daily (separate codes for battery error and patient user errors)

These were selected daily for each patient. Concurrently, improved systems for battery replacement have been implemented. Patient training has been provided when indicated. Alert thresholds have been updated as in cycle one.

**Cycle 2 result (due December):**

Early signs of reduced triage time and fewer red alerts will be quantified.

**Conclusions:**

Statistical analysis of measurement variation can provide a highly sensitive method for setting alert thresholds that accurately identifying illness however technical need reduced to improve specificity. The method is most effective for oxygen saturation. Improving effectiveness for other parameters may require improved patient training for equipment use (e.g. activities immediately prior to taking readings etc.). Research cycles using narrower standard deviations to improve specificity are required. If high sensitivity and specificity can be achieved, method can be used to automatically adjust thresholds periodically, reflecting the patient’s most recent clinical status.

